# SARS-CoV-2 501Y.V2 variants lack higher infectivity but do have immune escape

**DOI:** 10.1016/j.cell.2021.02.042

**Published:** 2021-04-29

**Authors:** Qianqian Li, Jianhui Nie, Jiajing Wu, Li Zhang, Ruxia Ding, Haixin Wang, Yue Zhang, Tao Li, Shuo Liu, Mengyi Zhang, Chenyan Zhao, Huan Liu, Lingling Nie, Haiyang Qin, Meng Wang, Qiong Lu, Xiaoyu Li, Junkai Liu, Haoyu Liang, Yi Shi, Yuelei Shen, Liangzhi Xie, Linqi Zhang, Xiaowang Qu, Wenbo Xu, Weijin Huang, Youchun Wang

**Affiliations:** 1 Division of HIV/AIDS and Sex-transmitted Virus Vaccines, Institute for Biological Product Control, National Institutes for Food and Drug Control (NIFDC) and WHO Collaborating Center for Standardization and Evaluation of Biologicals, No. 31 Huatuo Street, Daxing District, Beijing 102629, China; 2 CAS Key Laboratory of Pathogenic Microbiology and Immunology, Institute of Microbiology, Chinese Academy of Sciences (CAS), Beijing 100101, China; 3 Beijing Biocytogen Co., Ltd., Beijing 101111, China; 4 Beijing Antibody Research Key Laboratory, Sino Biological Inc., Building 9, Jing Dong Bei Technology Park, No. 18 Ke Chuang 10th St., BDA, Beijing 100176, China; 5 Center for Global Health and Infectious Diseases, Comprehensive AIDS Research Center, and Beijing Advanced Innovation Center for Structural Biology, School of Medicine, Tsinghua University, Beijing 100084, China; 6 Translational Medicine Institute, The First People’s Hospital of Chenzhou, University of South China, Chenzhou 423000, China; 7 National Institute for Viral Disease Control and Prevention, Chinese Center for Disease Control and Prevention, Beijing 102206, China

**Keywords:** SARS-CoV-2, mutation, receptor binding region, infectivity, neutralizing antibody, immune escape, 501Y.V2, N501Y, E484K, K417N

## Abstract

The 501Y.V2 variants of SARS-CoV-2 containing multiple mutations in spike are now dominant in South Africa and are rapidly spreading to other countries. Here, experiments with 18 pseudotyped viruses showed that the 501Y.V2 variants do not confer increased infectivity in multiple cell types except for murine ACE2-overexpressing cells, where a substantial increase in infectivity was observed. Notably, the susceptibility of the 501Y.V2 variants to 12 of 17 neutralizing monoclonal antibodies was substantially diminished, and the neutralization ability of the sera from convalescent patients and immunized mice was also reduced for these variants. The neutralization resistance was mainly caused by E484K and N501Y mutations in the receptor-binding domain of spike. The enhanced infectivity in murine ACE2-overexpressing cells suggests the possibility of spillover of the 501Y.V2 variants to mice. Moreover, the neutralization resistance we detected for the 501Y.V2 variants suggests the potential for compromised efficacy of monoclonal antibodies and vaccines.

## Highlights

• 501Y.V2 showed no higher infectivity in cells with hACE2 comparing to 614G variant
• 501Y.V2 showed increased infectivity in cells with mACE2 compared to 614G variant
• 501Y.V2 escaped neutralization by most of neutralizing monoclonal antibodies
• 501Y.V2 significantly compromised the inhibitory effects of polyclonal antibodies

## Graphical abstract

**Figure:**
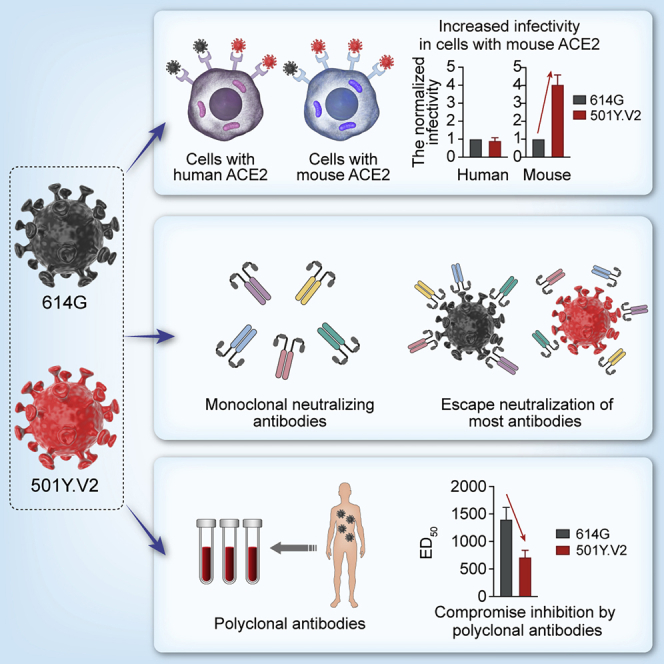

## Teaser

Experiments with pseudotyped viruses show that the 501Y.V2 variant of SARS-CoV-2 exhibits resistance to neutralization from monoclonal antibodies and sera from convalescent as well as immunized individuals, predominantly due to the E484K and N501Y mutations in the receptor-binding domain of the viral spike protein.

## Introduction

As of early February 2021, SARS-CoV-2 had infected more than 100 million people worldwide and killed more than 2 million people (https://covid19.who.int). SARS-CoV-2 is a member of the coronavirus family, which carries the largest genome among single-stranded RNA viruses. Although the replication-dependent RNA polymerase in most RNA viruses has no proofreading activity, the coronavirus genome encodes a 3′–5′ exonuclease (ExoN, nsp14) with proofreading activity that can partially correct mutations introduced during virus replication (Smith and Denison, 2013). Accordingly, coronaviruses mutate less frequently than other RNA viruses. Even so, there are now reports of multiple variants emerging around the world as the duration of the SARS-CoV-2 pandemic extends (Hodcroft et al., 2020; Kirby, 2021; Korber et al., 2020; Makoni, 2021; Tang et al., 2020, 2021).

Some mutations in the spike (S) protein of SARS-CoV-2 can increase the infectivity of the virus. For example, the D614G mutation in the S protein increases viral infectivity in susceptible cells by 8- to 10-fold (Li et al., 2020; Zhang et al., 2020), and both the infectivity and transmissibility of the D614G mutant virus are significantly elevated in a hamster model (Hou et al., 2020; Plante et al., 2020). This may at least partially explain how the 614G virus spread so rapidly; 614G overtook the 614D virus within 3 months of its emergence in February 2020 (Korber et al., 2020).

Fortunately, this 614G mutation did not cause a significant change in viral antigenicity that would allow its escape from immune responses resulting from infection with the original virus or from a vaccine (Weissman et al., 2021). However, the selective pressure from S-specific antibodies induced by SARS-CoV-2 infection could promote acquisition of additional mutations (e.g., in its N-terminal domain [NTD] and/or its receptor-binding domain [RBD]) that could lead to escape (Liu et al., 2020; Weisblum et al., 2020). Indeed, studies have identified multiple naturally occurring mutations that result in escape from multiple monoclonal antibodies and convalescent sera (Li et al., 2020; Thomson et al., 2021).

The Republic of South Africa currently has the highest numbers of SARS-CoV-2-infected cases and COVID-19-related deaths in Africa. The initial SARS-CoV-2 epidemic in South Africa primarily involved the B.1 lineage identified in Italy (Giandhari et al., 2021). The predominant variants in South Africa appear to be changing rapidly: in April, the first region-specific lineage, B.1.106, was detected in nosocomial infections in South Africa. On successful control of nosocomial infection, this viral lineage gradually disappeared (James et al., 2020). The first epidemic peak of SARS-CoV-2 in South Africa occurred from June to September, primarily driven by three lineages: B.1.1.54, B.1.1.56, and C.1 (Tegally et al., 2020b). The only reported difference in the S protein amino acid sequences between these lineages and the Wuhan-1 strain is the D614G mutation (Tegally et al., 2020b).

South Africa experienced a brief plateau following the first wave of the epidemic. However, the number of SARS-CoV-2 infections in South Africa has increased exponentially since mid-October of 2020. In this outbreak, a new 501Y.V2 lineage (also known as B.1.351) was identified; variants of this lineage are genetically distinct from those of the first wave. By early November, the number of new cases infected with the 501Y.V2 variants exceeded the total infections by all of the variants from the first wave of the epidemic. It has therefore been assumed that 501Y.V2 variants have become the predominant epidemic variants in South Africa (Tegally et al., 2020a).

In the present study, we refer to the three most prevalent variants of the 501Y.V2 lineages as 501Y.V2-1, 501Y.V2-2, and 501Y.V2-3. In the early stages of the second wave, 501Y.V2-1 was prevalent; it is identifiable by five amino acid mutations in the S protein (in addition to D614G), including D80A, D215G, E484K, N501Y, and A701V. Subsequently, two further mutations arose in the S protein, L18F, and K417N, resulting in the emergence of variant 501Y.V2-2. The third variant (501Y.V2-3) appeared based on deletion of S protein residues (Del242-244) from 501Y.V2-2. Compared against the S protein of the 614G virus shows that 501Y.V2-3’s S protein contains 8 mutations: four are located at the NTD (L18F, D80A, D215G, and Del242-244), three are in the viral RBD (K417N, E484K, and N501Y), and one is in the S2 region (A701V) (Tegally et al., 2020a).

In this communication, we investigated the biological significance—using assays of infectivity and of antigenicity—of a set of 18 501Y.V2 lineage-related mutants. Our approach was based on construction of 18 pseudotyped viruses using the vesicular stomatitis virus (VSV)-pseudovirus system, and we generated a pseudotyped reference 614G variant as a control for the assays. We analyzed the infectivity of the pseudotyped viruses for multiple SARS-CoV-2-susceptible cell lines and for a panel of HEK293T cells expressing the ACE2 ortholog proteins from a total of 14 mammal species. We also profiled the antigenicity of the pseudotyped viruses to monoclonal antibodies, to SARS-CoV-2 convalescent sera, and to RBD immunize animal sera. We found that the 501Y.V2 variants showed no increased infectivity for SARS-CoV-2-susceptible human cell lines; however, the 501Y.V2 variants were less susceptible to the neutralizing activity of antibodies compared to the 614G variant. Similar results have also been reported by Daming Zhou's group when tested 501Y.V2 against vaccine-induced and SARS-CoV-2-infected sera (Zhou et al., 2021).

## Results

### Construction of the pseudotyped viruses related to 501Y.V2

**Figure 1:**
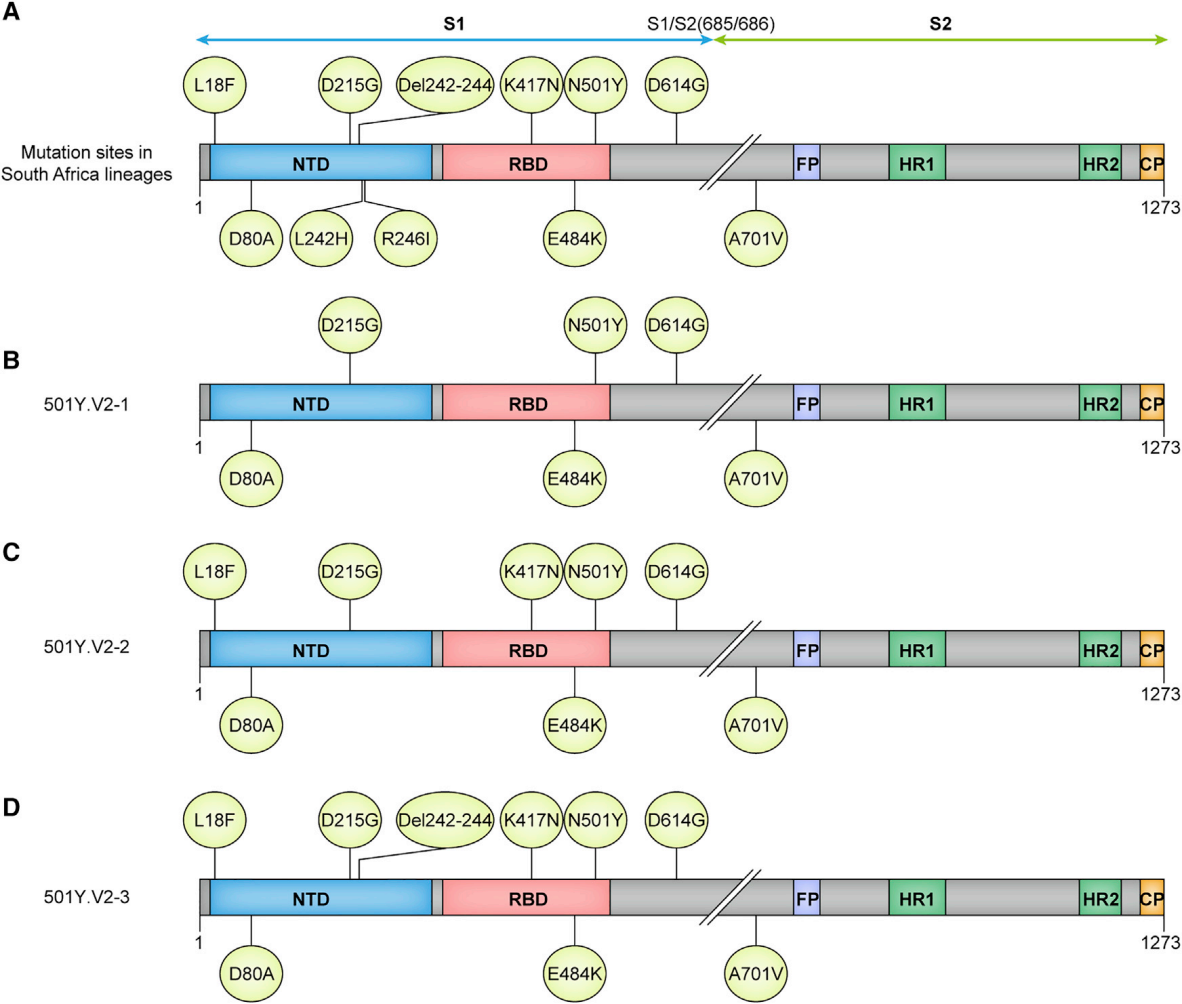
Illustration of 501Y.V2-related pseudotyped viruses (A) All the 501Y.V2-related mutation sites. (B) 501Y.V2-1. (C) 501Y.V2-2. (D) 501Y.V2-3.

To study the effects of 501Y.V2-related mutations, we generated a total of 18 pseudotyped viruses. The 501Y.V2 variants, derived from B.1 (Tegally et al., 2020a), have the D614G S protein mutation. Note that all of the pseudotyped viruses in this study were generated in the 614G background using site-directed mutagenesis, and 614G was used as the reference pseudotyped virus for our experimental infectivity assays with diverse host cells and antigenicity assays with various antibodies and sera. We first constructed a set of 10 pseudotyped viruses carrying the single-site mutations in 501Y.V2 variants in a 614G background (Figure 1A). Then, we generated the three main variants, 501Y.V2-1, 501Y.V2-2, and 501Y.V2-3 (Figures 1B–1D). It is now clear that the SARS-CoV-2 RBD is an essential region for virus binding to the cell receptor ACE2 (Barnes et al., 2020; Hoffmann et al., 2020; Lan et al., 2020; Shang et al., 2020; Walls et al., 2020), and the RBD is also a dominant immune epitope of the S protein (Baum et al., 2020; Brouwer et al., 2020; Cao et al., 2020; Lv et al., 2020; Shi et al., 2020; Wu et al., 2020). 501Y.V2-3, which has three mutated amino acids in its RBD, is one of the most complicated SARS-CoV-2 variants detected to date (Tegally et al., 2020a). To help determine whether any epistatic and/or synergistic effects were conferred alongside the emergence of these three mutations in the RBD, we also constructed a total of four pseudotyped viruses carrying double or triple RBD mutations. We thus obtained the 18 pseudotyped viruses (including 614G variant) collectively representing the 501Y.V2-related mutations.

### Infectivity of the 501Y.V2-related variants

**Figure 2:**
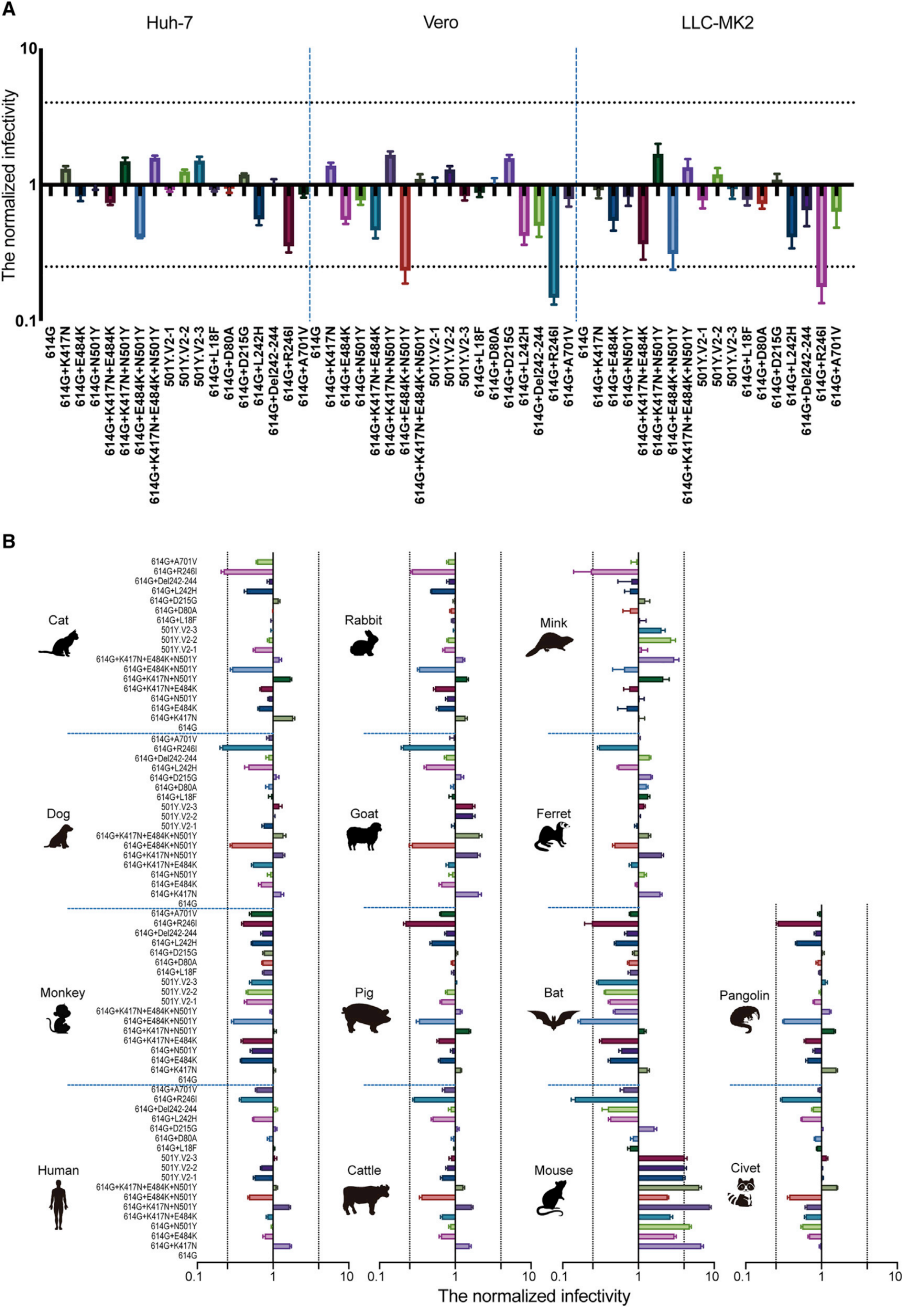
Infectivity analysis of mutant pseudotyped viruses (A) Infection assays with the 18 501Y.V2-related mutant pseudotyped viruses with the three indicated cell lines, all of which are known to be susceptible to SARS-CoV-2. (B) Infection assays for a set of 14 HEK293T cell lines each expressing the indicated mammalian ortholog of ACE2. The infectivity of the reference 614G variant was used as a control (i.e., the infectivity of other 17 variants in each experiment was normalized to values detected for the reference 614G variant). Data are the means ± SEM of six independent experiments. The dashed lines indicate the threshold value of a 4-fold difference in infectivity. See also Figure S1.

**Figure S1:**
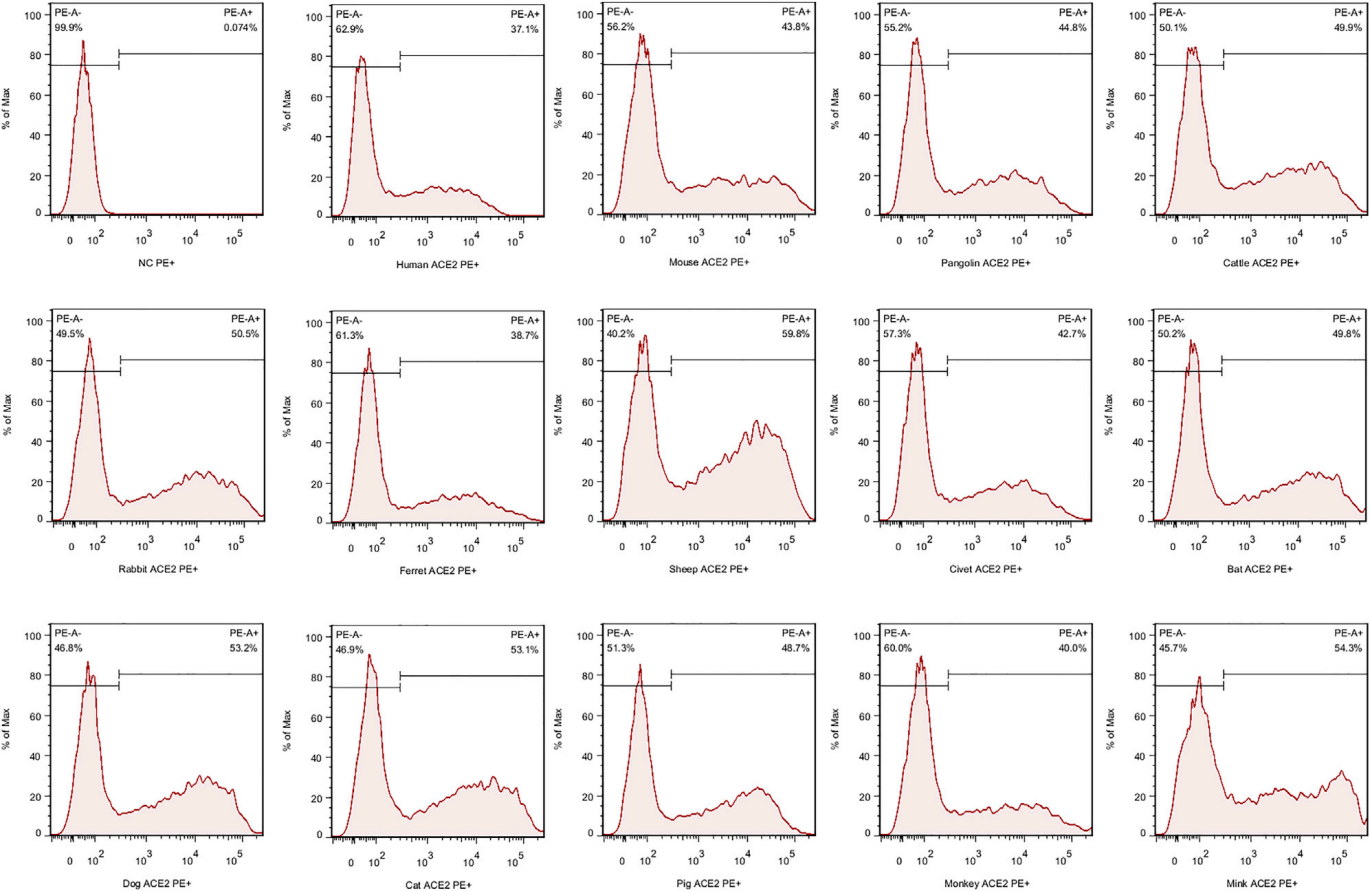
The expression levels of the various mammalian ACE2 orthologs on the surface of transfected HEK293T cells, related to Figure 2 The cell surface expression of the FLAG-tagged ACE2 orthologs was assessed by flow cytometry. The PE-A+ value in the upright corner represents the percentage of ACE2-expressing cells.

We first investigated the potential infection-related effects of these mutations in assays with three cell lines known to be susceptible to SARS-CoV-2 pseudotyped virus infection (Li et al., 2020): Huh-7, Vero, and LLC-MK2. Compared to the reference 614G variant, no significant increase in infectivity was observed in these cell lines for any of the pseudotyped viruses with 501Y.V2-related mutations (Figure 2A). We next characterized the infectivity of these pseudotyped viruses for cells expressing receptors from a diverse group of mammal species. Specifically, we used HEK293T cells transfected with individual plasmids containing the ACE2 genes from 14 species (all with FLAG-tags). ACE2 expression was monitored using flow cytometry: the percentage of ACE2-positive cells fell in a range of 37.1%–59.8% among these 14 cells (Figure S1).

We then challenged these ACE2 receptor expressing cells with our 18 pseudotyped viruses. The infectivity of individual variant in each ACE2 expressing cells was assessed relative to the reference 614G variant’s infectivity. For 13 of the 14 tested ACE2-expressing cells, no significant enhancement in infectivity were detected for any of the pseudotyped viruses with 501Y.V2-related mutations (Figure 2B). The exception here was the significant differences in infectivity observed with the HEK293T cells expressing murine ACE2. Three single-residue variants, K417N, E484K, and N501Y—all located at the RBD region—respectively displayed 7-fold, 3-fold, and 5-fold increases in infectivity compared to the reference 614G variant (Figure 2B). Moreover, the pseudotyped viruses carrying double (K417N+N501Y) and triple (K417N+E484K+N501Y) mutations exhibited yet-higher increases in infectivity compared to the single mutants (Figure 2B). Note that the pseudotyped viruses representing the three most prevalent variants (501Y.V2-1, 501Y.V2-2, and 501Y.V2-3) each had an ∼4-fold increase in infectivity in the murine ACE2-expressing cells (Figure 2B).

### Significantly decreased antigenicity of 501Y.V2 variants with monoclonal neutralizing antibodies

**Figure 3:**
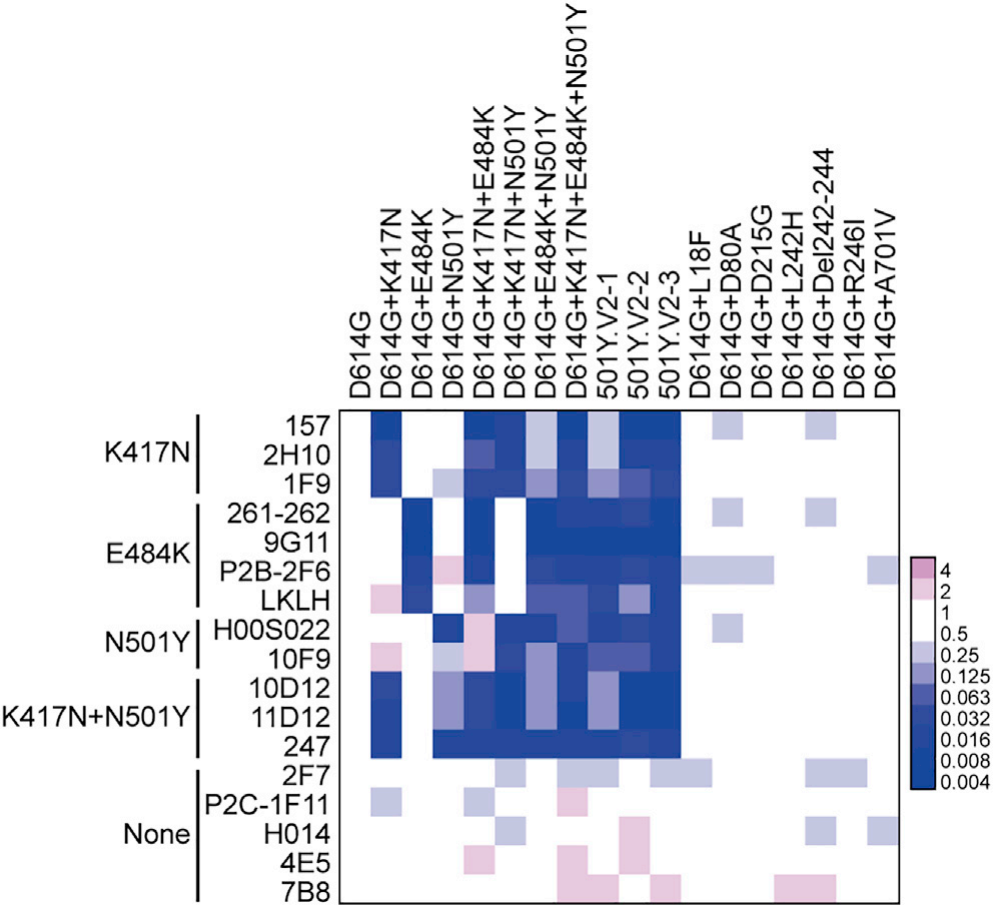
Analysis of antigenicity of 501Y.V2 variants using a panel of neutralizing monoclonal antibodies Heatmap representation of neutralization reactions using 17 neutralizing monoclonal antibodies—all known to target epitopes in the RBD—against 18 501Y.V2-related mutant pseudotyped viruses; the ratio of EC_50_ value (for each of the tested antibodies) detected for the reference 614G variant to the EC_50_ value for each of 501Y.V2-related mutant pseudotyped viruses. Blue and pink represent decreased and increased viral sensitivity to monoclonal antibody neutralization, respectively. Data represent the means of three independent experiments. See also Figures S2 and S3.

**Figure S2:**
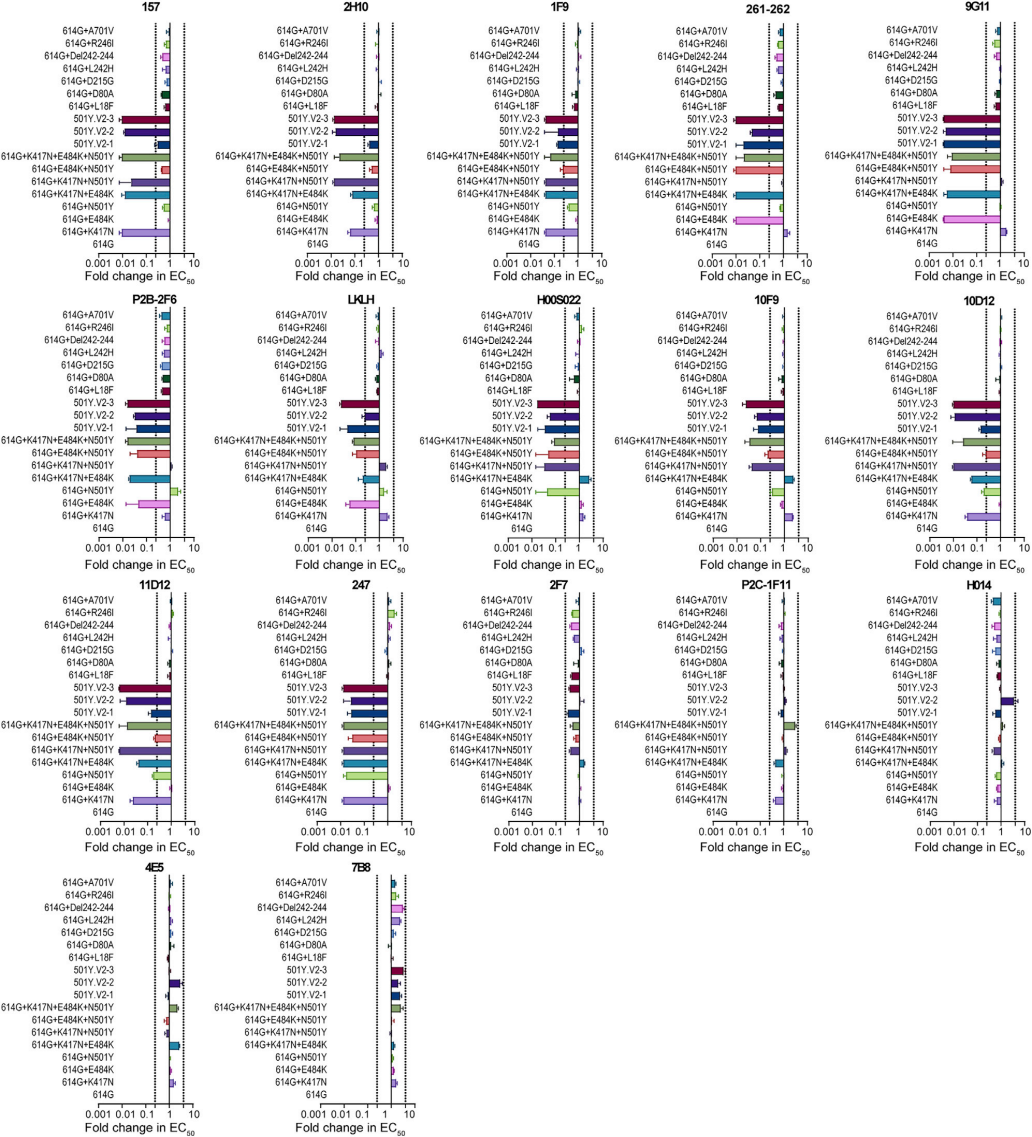
Reactivity of pseudotyped viruses with 501Y.V2 related mutations to 17 neutralized monoclonal antibodies, related to Figure 3 The data represent the ratio of the EC_50_ value for the reference 614G pseudotyped virus to the pseudotyped viruses harboring 501Y.V2 related mutations. Data represent the the means of three independent experiments. The dashed line indicates the threshold value of a 4-fold difference in EC_50_.

**Figure S3:**
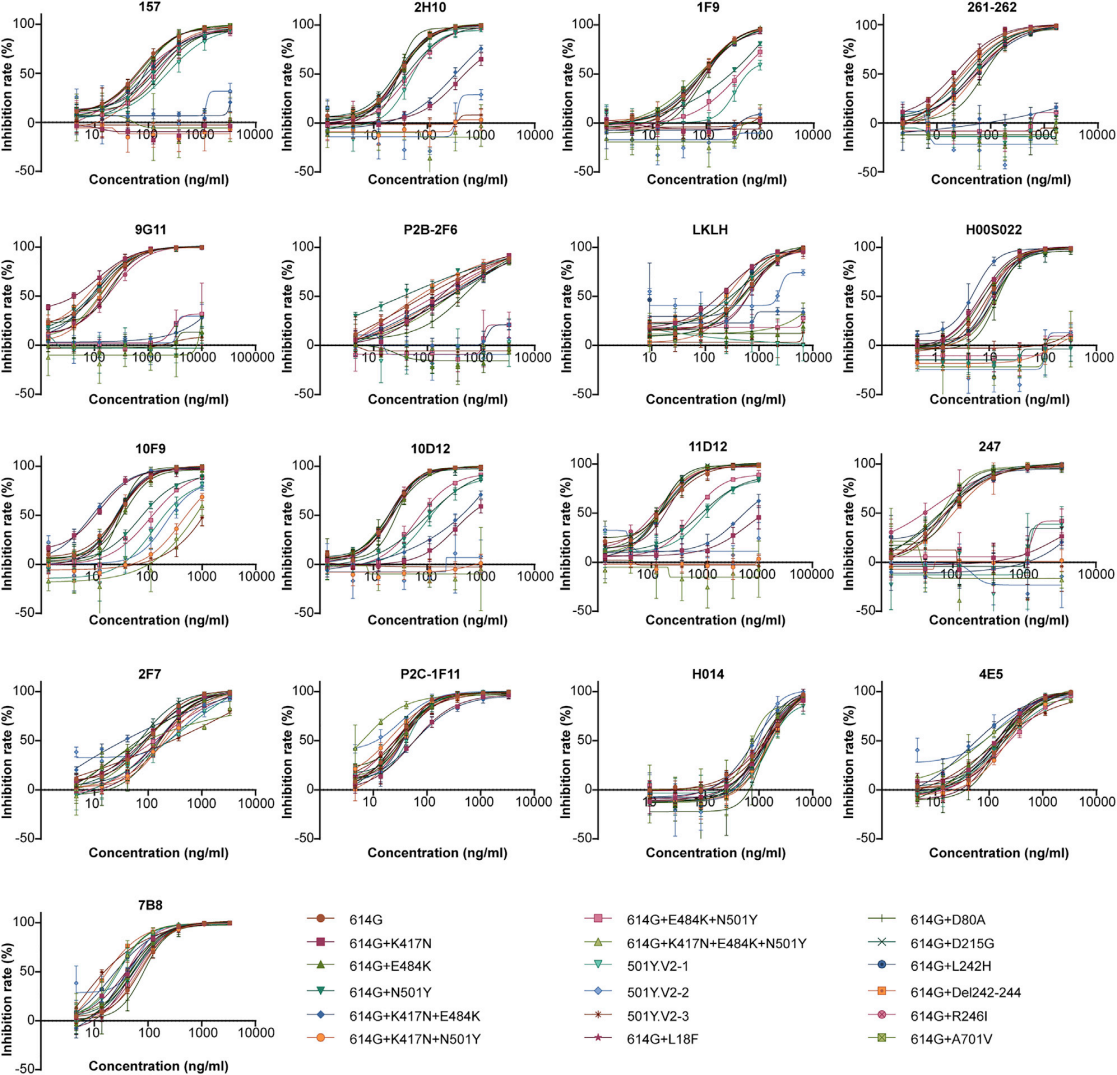
Neutralization curves of the 17 neutralized monoclonal antibodies against the pseudotyped viruses with 501Y.V2 related mutations, related to Figure 3

To study the effects of 501Y.V2-related mutations on viral antigenicity we tested our 18 pseudotyped viruses against a set of 17 neutralizing monoclonal antibodies targeting the RBD. Strikingly, most of monoclonal antibodies used in this study showed decreased neutralizing activity to the pseudotyped viruses carrying mutations in the RBD compared to the reference 614G variant (Figures 3, S2, and S3). By defining immune escape as a 4-fold decrease in neutralizing activity of a monoclonal antibody compared to the reference 614G variant, we divided the 17 monoclonal antibodies into five groups based on mutation sites. Briefly, escape from the 157, 2H10, and 1F9 antibodies was caused by the K417N mutation; escape from 261-262, 9G11, P2B-2F6, and LKLH was caused by the E484K mutation; escape from H00S022 and 10F9 was caused by the N501Y mutation; and escape from 10D12, 11D12, and 247 was caused by both K417N and N501Y (Figure 3). No alteration of neutralization sensitivity was observed for 5 of the 17 monoclonal antibodies (2F7, P2C-1F11, H014, 4E5, and 7B8).

We found that an increasing number of mutation sites in the RBD was correlated with immune escape from a steadily increasing number of monoclonal antibodies (Figure 3), clearly suggesting a superposition effect. Conversely, monoclonal antibodies that do not neutralize any of the three RBD site mutations were also ineffective in neutralizing the 501Y.V2 variants containing those mutations. 501Y.V2-1 was a relatively early variant in the second wave of this epidemic (Tegally et al., 2020a); it carries the E484K and N501Y mutations but not the K417N mutation. We found that the antibody escape spectrum of our pseudotyped virus 501Y.V2-1 was essentially the same as for the 614G+E484K+N501Y triple RBD mutation variants. However, and recalling that the 501Y.V2-2 pseudotyped virus carries two additional mutations (L18F and K417N), it is consistent that 501Y.V2-2’s escape spectrum is wider than 501Y.V2-1’s spectrum for this panel of neutralizing antibodies (Figure 3). Finally, our finding that 501Y.V2-3’s escape spectrum for this RBD-targeting antibody panel is identical to 501Y.V2-2’s spectrum fit with our expectations, because these two pseudotyped variants contain the same mutations in their RBDs (Figure S2).

### Altered reactivity of 501Y.V2 pseudotyped viruses with polyclonal antibodies

**Figure 4:**
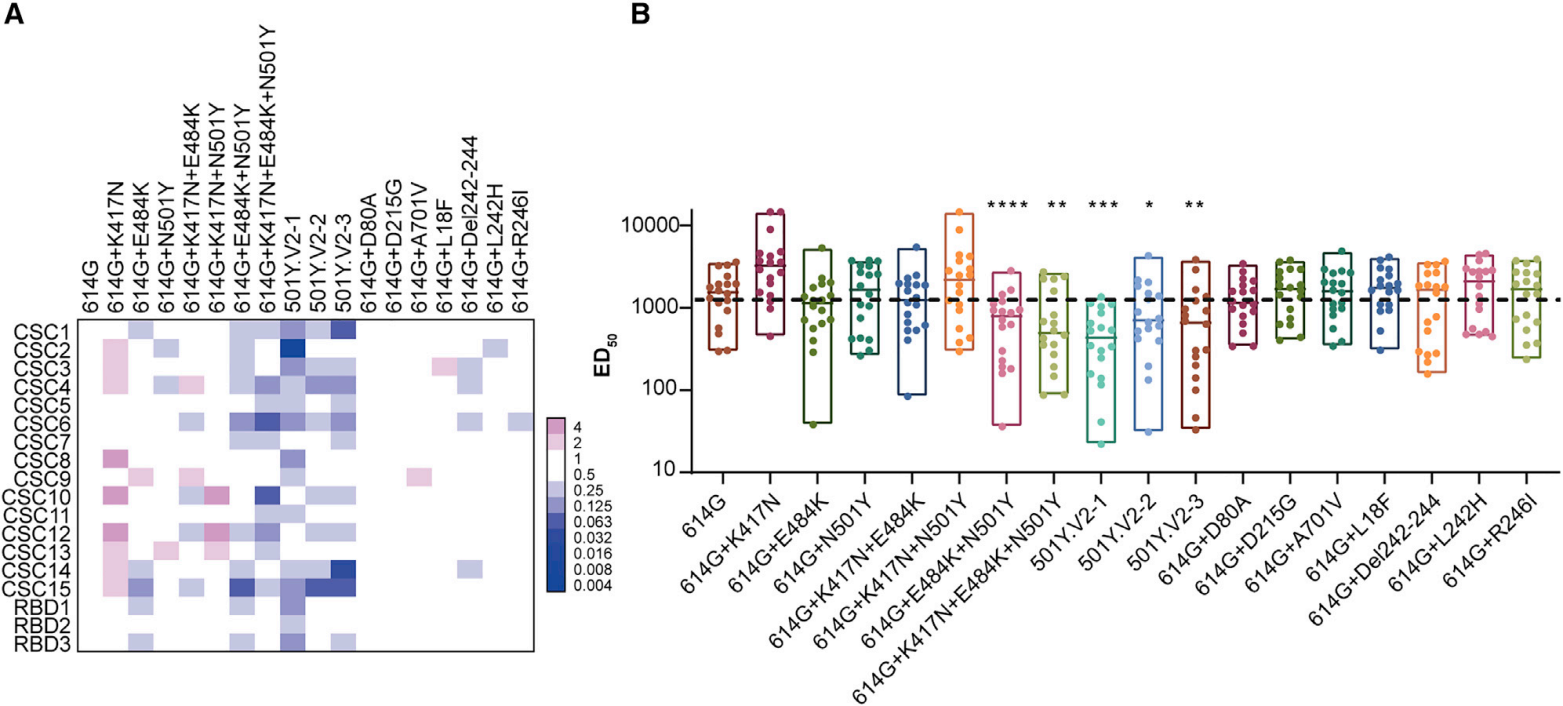
Analysis of antigenicity of 501Y.V2 variants using a panel of polyclonal antibodies (A) The reactivity of pseudotyped viruses with 501Y.V2-related mutations was assayed against sera from convalescent sera with high-titer polyclonal neutralizing antibodies (“CSC”) from SARS-CoV-2 infection patients and against 3 pooled sera samples (from 9 mice were immunized with RBD [“RBD”]). The data (means of three independent experiments) presented in the heatmap show the ratio of the ED_50_ value detected for each of the 501Y.V2-related mutant pseudotyped viruses to the value detected for the reference 614G virus. Blue and pink represent decreased and increased viral sensitivity to neutralization by sera, respectively. (B) Summary and inferential statistical analysis of the results for the pseudotyped viruses with 501Y.V2-related mutations. The dashed line represents the mean serum response of the 614G virus. One-way ANOVA and Holm-Sidak’s multiple comparison tests were used to analyze the differences between groups. A p value of <0.05 was considered to be significant. ^∗^p < 0.05, ^∗∗^p < 0.01, ^∗∗∗^p < 0.001.

We also obtained convalescent sera from 15 SARS-CoV-2-infected patients with high neutralizing antibody titers and obtained three pooled sera samples from a total of nine mice immunized with the RBD to further investigate how these mutations affect antigenicity (Figure 4A). Neutralization assays with the pseudotyped viruses showed that mutations at a single site did not lead to significant alteration of the neutralization activity of polyclonal antibodies; only the simultaneous presence of the E484K and N501Y mutations resulted in a significant decrease in neutralization (p < 0.05) (Figure 4B). Among the 501Y.V2 pseudotyped viruses, 501Y.V2-1 showed the greatest decrease in neutralization by polyclonal antibodies, displaying a 3.9-fold reduction compared to the reference 614G variant (Figure 4B). Note that 501Y.V2-1 lacks the K417N mutation, so it appears that for 501Y.V2-2 and for 501Y.V2-3, the presence of K417N apparently increases susceptibility to neutralization by polyclonal antibodies.

**Figure 5:**
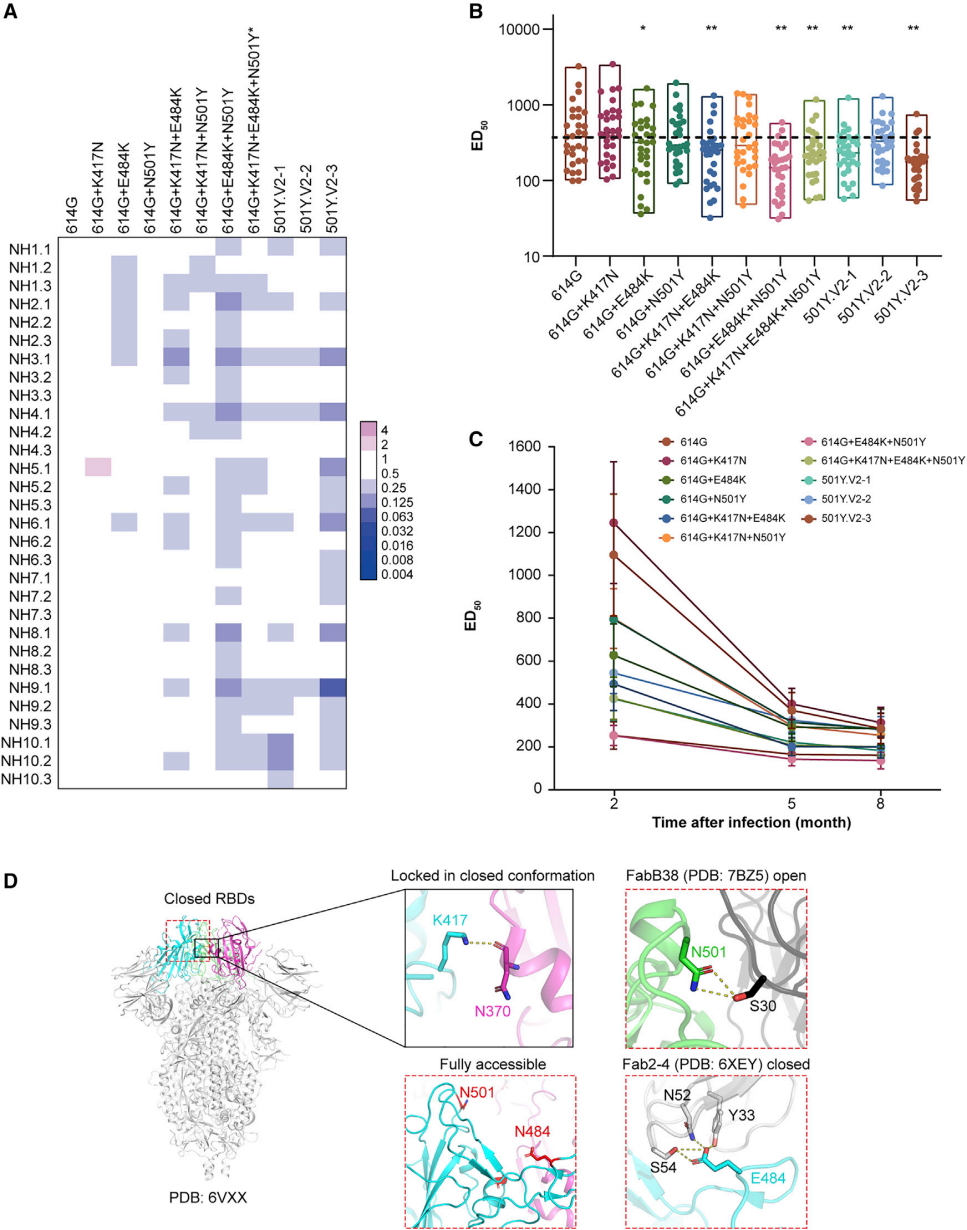
Testing of longitudinal convalescent sera samples obtained from SARS-CoV-2 infected patients at 2, 5, and 8 months post-onset (A) Heatmap analysis of the ratios of ED_50_ values of pseudotyped viruses with 501Y.V2-related mutations to the reference 614G virus. Blue and pink represent decreased and increased viral sensitivity to neutralization by sera, respectively. Data represent the means of two independent experiments. (B) Summary and inferential statistical analysis of the results of different mutants. The dashed line represents the mean serum response of the reference 614G virus. One-way ANOVA and Holm-Sidak’s multiple comparisons test were used to analyze the differences between groups. A p-value of <0.05 was considered to be significant. ^∗^p < 0.05, ^∗∗^p < 0.01. (C) Analysis of the results of reactions between 501Y.V2-related mutant pseudotyped viruses with longitudinal sera from SARS-CoV-2 infection patients at 2, 5, and 8 months post onset. (D) Model of the S protein trimer (PDB: 6VXX) with human ACE2 and neutralizing antibodies (PDB: 7BZ5, 6XEY). K417 forms hydrogen bonds with the main chain of N370 in the neighboring S protomer in the closed conformation of the S protein.

To determine how the mutations in the 501Y.V2 variants may affect neutralization activity in the sera with differing levels of neutralizing antibodies, we obtained longitudinal sera from ten SARS-CoV-2-infected patients at 2, 5, and 8 months after onset (Figure 5A). The pseudotyped viruses with 501Y.V2-related RBD mutations and the 614G control virus then were used in assays with these 30 longitudinal sera samples. We found that the E484K and N501Y mutations led to a decrease in neutralization, and the combination of these two mutations resulted in an apparently superimposed resistance to neutralization (Figure 5B). Further, it was again conspicuous that the K417N mutation increased viral susceptibility to neutralization.

Taking the reference 614G pseudotyped virus as an example: compared to assays for the sera collected at 2 months, neutralization titers for sera collected at 5- and 8-months post-onset decreased by 2.2- and 2.5-fold, respectively (Figure 5C). We noted that the trends for detected decreases varied consistently within sera of differing antibody titers: the higher the antibody titer, the greater the reduction in the neutralizing activity (Figure 5C). The most pronounced differences from the reference 614G pseudotyped virus were detected for 501Y.V2-3, which exhibited reduced neutralization at antibody titers >1,000, 500–1,000, and <500 by an average of 5.3-, 3.1-, and 1.8-fold, respectively. Some samples with low antibody titers (median effect dose, ED_50_ for 614G <100) were not able to neutralize 501Y.V2-3 (ED_50_ <30).

## Discussion

Mutation is a common phenomenon in the natural evolution of viruses, and SARS-CoV-2 is no exception. The emergence of a variety of SARS-CoV-2 mutants has become a major concern during the ongoing pandemic. Mutants may be more transmittable, or may be able to evade neutralizing monoclonal antibodies, or even polyclonal antibodies induced by either infection or vaccination. That is, a shift in the predominant variant(s) in various epidemics could cause potentially declines in the protective effects of vaccines or neutralizing monoclonal antibodies that were developed based on the original variant. Here, we constructed 18 501Y.V2-related pseudoviruses using a VSV-based system and systematically studied the effects of mutations on virus infectivity and antigenicity. We found that, compared with the reference 614G variant, the infectivity of the 501Y.V2 variants in human receptor cells did not change significantly, but did alter antigenicity. The neutralizing activity of multiple RBD-targeting monoclonal antibodies decreased significantly, and polyclonal antibodies (from RBD-immunized mouse sera and from SARS-CoV-2 convalescent sera) also had decreased neutralizing activity against 501Y.V2 variants to certain degrees.

Previous reports have shown that passage of SARS-CoV-2 in mice can result in an increase in infectivity toward mice and can cause symptoms similar to human COVID-19, including interstitial pneumonia and inflammatory responses (Gu et al., 2020). This enhanced adaptation to murine hosts is at least partially attributable to the occurrence of the N501Y mutation (Gu et al., 2020). With increasing numbers of SARS-CoV-2 passages in mice, the virulence of the virus also increases, eventually leading to the generation of variants that can cause death in mice (Sun et al., 2020). The lethal variant is characterized by the superposition of two RBD mutations, Q493H and K417N, in the N501Y mutant background. The superposition of each successive mutation further enhances the S protein affinity to murine ACE2, consequently leading to increased virulence in mice (Sun et al., 2020). We found that multiple pseudotyped viruses harboring N501Y and K417N mutations (including 501Y.V2-2 and 501Y.V2-3) were significantly more infective toward HEK293T cells expressing murine ACE2 compared to the reference 614G variant. At minimum, these findings suggest a risk that the predominant variants of the 501Y.V2 lineage could be transmitted to mice, further extending the SARS-CoV-2 host range.

Monoclonal antibodies P2C-1F11 and H014 showed no reduction in their neutralizing capacity against all three 501Y.V2 pseudotyped viruses. The common feature of these two antibodies is that they both have a relatively high number of binding sites within the RBD. The binding interface between P2C-1F11 and RBD involves 22 amino acid residues (Ge et al., 2021). The RBD binding surface for H014 is even larger; all 6 complementary determinants of the antibody (CDRL1-3 and CDRH1-3) are involved, enabling this antibody to cross-neutralize SARS-CoV and SARS-CoV-2 (Lv et al., 2020). By contrast, the RBD binding surface with P2B-2F6, which cannot neutralize variants carrying the E484K mutation, includes only 14 residues (Ge et al., 2021). The high binding affinity of P2C-1F11 (Ge et al., 2021) suggests the following: viral mutations are less likely to compromise the potency of those monoclonal antibodies that engage with more residues in RBD.

Although H014 and P2C-1F11 neutralized all of the 501Y.V2 variants we tested in the present study, we previously showed that some other mutations in the RBD region lead to decreased neutralizing capability for P2C-1F11 (A475V) and H014 (A435S and Y508H) (Li et al., 2020). Thus, given the negative effect of continuous viral variation on antibody potency, monoclonal antibodies used in cocktails for preventive or treatments of SARS-CoV-2 would ideally incorporate a large binding area, high binding affinity, and a wide variety of binding epitopes to ensure maximum possible efficacy in neutralization while also retaining the broadest achievable spectrum against immune escape.

Interestingly, our data from assays with convalescent sera indicate that the K417N mutation actually increases viral sensitivity to neutralization. Consider that in the closed conformation of the S protein, K417 forms hydrogen bonds with the main chain of N370 in the neighboring S protomer (Figure 5D), resulting in stabilization; the closed conformation structure does not readily bind to ACE2 (Walls et al., 2020) and presents a reduced overall area accessible to antibodies. The K417N mutation increases the probability of conversion to the open conformation, thus enhancing the S protein’s binding capacity for ACE2 and increasing viral infectivity. This closed-to-open change in the S protein’s conformation is also more likely to expose epitopes to neutralizing antibodies, which would increase the likelihood of virus neutralization by sera containing polyclonal antibodies. Because both the E484 and N501 residues are fully exposed, it is reasonable to speculate that mutations to these sites may weaken antibody binding (Figure 5D), potentially thereby reducing the sensitivity of the virus to neutralizing antibodies.

It is notable that residue 484 has mutated into a variety of different amino acids under pressure of SARS-CoV-2 convalescent sera (Liu et al., 2020) (e.g., E484A, E484G, E448D, and E484K), and mutation at this site can cause immune resistance to different convalescent sera (Liu et al., 2020). This variability indicates that 484E is located at a “dominant epitope region” of the S protein. All variants of 501Y.V2 harbor the E484K mutation, further supporting that this mutation can at least partially explain the observed decreased susceptibility to neutralization by convalescent sera.

The 501Y.V2 variants showed no obvious changes in infectivity SARS-CoV-2-susceptible cell lines. However, RBD mutations led to significantly higher viral infection in HEK293T cells expressing the murine ortholog of ACE2. Simultaneous mutation of three amino acids in the RBD of the 501Y.V2 variants decreased sensitivity to neutralization by SARS-CoV-2 convalescent sera and RBD-immunized sera, while mutations outside of the RBD had minimal effects on viral infectivity and antigenicity. Moreover, our data support that the predominant 501Y.V2 variants may compromise the therapeutic efficacy of existing monoclonal antibodies or convalescence sera, or even cause a decrease in the protective efficacy of existing vaccines. Therefore, studies on SARS-CoV-2 reinfection should also be conducted to evaluate whether the immune response established by an early viral infection can prevent reinfection by the newer mutant variants. It also remains unclear whether the variants induce strong immune responses. Close monitoring and functional genetic analysis of these prevalent variants could be informative for guiding prevention and control measures for the SARS-CoV-2 pandemic.

### Limitations of study

The application of pseudotyped virus to study the infectivity and antigenicity of the virus has been widely used in the field of virus research (Ferrara and Temperton, 2018; Li et al., 2018; Whitt, 2010), especially in the study of SARS-CoV-2 (Crawford et al., 2020; Lei et al., 2020; Li et al., 2020; Nie et al., 2020a, 2020b; Schmidt et al., 2020; Weissman et al., 2021; Zheng et al., 2020). Nevertheless, it should be noted that all the results of this study are based on assays using pseudotyped viruses. That is, there is as yet no verification of the detected trend from experiments using the live virus. It is difficult to obtain live mutant virus variants. In particular, it is not possible to obtain certain strains we examined based on isolating live viruses (e.g., virus strains with 501Y.V2-related single-mutations or some of the different combinations of RBD mutation sites). Another limitation of our study is that we did not examine immune sera from individuals who had received licensed or candidate vaccines. Exploring the potential for differential neutralization effects for the 501Y.V2 variants with vaccine immune sera could extend our findings and help in public health planning.

## STAR★Methods

### Key resources table

**Table:** 

**REAGENT or RESOURCE**	SOURCE	IDENTIFIER
**Monoclonal antibodies**		

H014	Sino Biological Company; (Lv et al., 2020)	N/A
H00S022	Sino Biological Company	N/A
P2C-1F11	Laboratory of Linqi Zhang; (Ju et al., 2020)	N/A
P2B-2F6	Laboratory of Linqi Zhang; (Ju et al., 2020)	N/A
261-262	Laboratory of Linqi Zhang	N/A
157	Laboratory of Linqi Zhang	N/A
247	Laboratory of Linqi Zhang	N/A
1F9	Biocytogen Inc.	N/A
7B8	Biocytogen Inc.	N/A
4E5	Biocytogen Inc.	N/A
2F7	Biocytogen Inc.	N/A
2H10	Biocytogen Inc.	N/A
10D12	Biocytogen Inc.	N/A
10F9	Biocytogen Inc.	N/A
11D12	Biocytogen Inc.	N/A
LKLH	Biocytogen Inc.	N/A
9G11	Biocytogen Inc.	N/A

**Bacterial and virus strain**		

DH5α Chemically Competent Cell	Invitrogen	Cat.# 12034013
G^∗^ΔG-VSV	Kerafast	Cat.# EH1020-PM

**Biological samples**		

Convalescent patient serum, CSC1	This paper	N/A
Convalescent patient serum, CSC2	This paper	N/A
Convalescent patient serum, CSC3	This paper	N/A
Convalescent patient serum, CSC4	This paper	N/A
Convalescent patient serum, CSC6	This paper	N/A
Convalescent patient serum, CSC7	This paper	N/A
Convalescent patient serum, CSC8	This paper	N/A
Convalescent patient serum, CSC9	This paper	N/A
Convalescent patient serum, CSC10	This paper	N/A
Convalescent patient serum, CSC11	This paper	N/A
Convalescent patient serum, CSC12	This paper	N/A
Convalescent patient serum, CSC13	This paper	N/A
Convalescent patient serum, CSC14	This paper	N/A
Convalescent patient serum, CSC15	This paper	N/A
Convalescent patient serum, NH1.1	This paper	N/A
Convalescent patient serum, NH1.2	This paper	N/A
Convalescent patient serum, NH1.3	This paper	N/A
Convalescent patient serum, NH2.1	This paper	N/A
Convalescent patient serum, NH2.2	This paper	N/A
Convalescent patient serum, NH2.3	This paper	N/A
Convalescent patient serum, NH3.1	This paper	N/A
Convalescent patient serum, NH3.2	This paper	N/A
Convalescent patient serum, NH3.3	This paper	N/A
Convalescent patient serum, NH4.1	This paper	N/A
Convalescent patient serum, NH4.2	This paper	N/A
Convalescent patient serum, NH4.3	This paper	N/A
Convalescent patient serum, NH5.1	This paper	N/A
Convalescent patient serum, NH5.2	This paper	N/A
Convalescent patient serum, NH5.3	This paper	N/A
Convalescent patient serum, NH6.1	This paper	N/A
Convalescent patient serum, NH6.2	This paper	N/A
Convalescent patient serum, NH6.3	This paper	N/A
Convalescent patient serum, NH7.1	This paper	N/A
Convalescent patient serum, NH7.2	This paper	N/A
Convalescent patient serum, NH7.3	This paper	N/A
Convalescent patient serum, NH8.1	This paper	N/A
Convalescent patient serum, NH8.2	This paper	N/A
Convalescent patient serum, NH8.3	This paper	N/A
Convalescent patient serum, NH9.1	This paper	N/A
Convalescent patient serum, NH9.2	This paper	N/A
Convalescent patient serum, NH9.3	This paper	N/A
Convalescent patient serum, NH10.1	This paper	N/A
Convalescent patient serum, NH10.2	This paper	N/A
Convalescent patient serum, NH10.3	This paper	N/A

**Chemicals**		

PrimeSTAR	Takara	Cat.# R040A
DpnI	NEB	Cat.# R0176S
PE-anti-Flag antibody	Biolegend	Cat.# 637310
QIAamp Viral RNA Mini Kit	QIAGEN	Cat.# 52906
SuperScript III First-Strand Synthesis System for RT-PCR kit reagent	Invitrogen	Cat.# 18080-051
TB Green Premix Ex TaqII	Takara	Cat.# RR820A
Lipofectamine 2000 Transfection Reagent	Thermo Fisher Scientific	Cat.# 11668019
Lipofectamine 3000 Transfection Reagent	Thermo Fisher Scientific	Cat.# 2041107
Dulbecco’s modified Eagle medium (high glucose)	Hyclone	Cat.# SH30243.01
Penicillin-Streptomycin solution	GIBCO	Cat.# 15140163
20 mM N-2-hydroxyethyl piperazine-N-2-ethane sulfonic acid	GIBCO	Cat.# 15630080
fetal bovine serum(FBS,Pansera ES)	PAN-Biotech GmbH	Cat.# ST30-2602
Trypsin-EDTA (0.25%)	GIBCO	Cat.# 25200056

**Critical commercial assays**		

Britelite plus reporter gene assay system	PerkinElmer	Cat.# 6066769

**Experimental models: Cell lines**		

293T	ATCC	CRL-3216, RRID: CVCL_0063
Huh-7	JCRB	0403, RRID: CVCL_0336
Vero	ATCC	CCL-81, RRID: CVCL_0059
LLC-MK2	ATCC	CCL-7, RRID: CVCL_3009

**Deposited data**		

Repository of raw data	This paper	http://dx.doi.org/10.17632/hkg5wjv9ry.2

**Oligonucleotides**		

VSV (P protein)-F:TCTCGTCTGGATCAGGCGG	Sangon Biotech	N/A
VSV (P protein)-R: TGCTCTTCCACTCCA GENEWIZ TCCTCT TGG	Sangon Biotech	N/A
L18F-F: TGGTGAGCAGCCAGTGCGTGAATTTCACCACCAG AACCCAGC	Sangon Biotech	N/A
D80A-F: CAATGGCACCAAGAGATTCGCCAATCCTGTGCTG CCTTTCAAT	Sangon Biotech	N/A
D215G-F: CACCCATTAATCTGGTGAGAGGCCTGCCTCAGG GCTTCAGC	Sangon Biotech	N/A
Del242-244F: TATCACCAGATTCCAGACCCTGCACAGATCATAT CTTACACC	Sangon Biotech	N/A
L242H-F: CACCAGATTCCAGACCCTGCATGCCCTGCACAG ATCATATC	Sangon Biotech	N/A
R246I-F: GACCCTGCTGGCCCTGCACATATCATATCTTACAC CAGGCGAT	Sangon Biotech	N/A
K417N-F: CGCCAGGGCAGACCGGCAATATCGCCGACTA CAATTAC	Sangon Biotech	N/A
E484K-F: CACCGTGTAATGGCGTGAAGGGCTTCAATTGC TACTTCC	Sangon Biotech	N/A
N501Y-F: AGAGCTACGGCTTCCAGCCTACCTACGGCGT GGGCTACCAGCCTTACAG	Sangon Biotech	N/A
D614G-F: GTGGCCGTGCTGTACCAGGGCGTGAATTGC ACCGAGGT	Sangon Biotech	N/A
A701V-F: CTACACCATGAGCCTGGGCGTGGAGAATAGC GTGGCCTAC	Sangon Biotech	N/A

**Recombinant DNA**		

Plasmid:pcDNA3.1.S2 (codon-optimized S gene of SARS-CoV-2, GenBank: MN908947)	Addgene	Addgene ID: 149457
Plasmid:pcDNA3.1.S2-D614G	This paper	N/A
Plasmid:pcDNA3.1.S2-D80A	This paper	N/A
Plasmid:pcDNA3.1.S2-D215G	This paper	N/A
Plasmid:pcDNA3.1.S2-E484K	This paper	N/A
Plasmid:pcDNA3.1.S2-N501Y	This paper	N/A
Plasmid:pcDNA3.1.S2-A701	This paper	N/A
Plasmid:pcDNA3.1.S2-L18F	This paper	N/A
Plasmid:pcDNA3.1.S2-K417N	This paper	N/A
Plasmid:pcDNA3.1.S2-Del242-244	This paper	N/A
Plasmid:pcDNA3.1.S2-K417N+ E484K	This paper	N/A
Plasmid:pcDNA3.1.S2-K417N+N501Y	This paper	N/A
Plasmid:pcDNA3.1.S2-E484K+N501Y	This paper	N/A
Plasmid:pcDNA3.1.S2-K417N+ E484K+N501Y	This paper	N/A
Plasmid:pcDNA3.1.S2-501Y.V2-1	This paper	N/A
Plasmid:pcDNA3.1.S2-501Y.V2-2	This paper	N/A
Plasmid:pcDNA3.1.S2-501Y.V2-3	This paper	N/A
Plasmid:pcDNA3.1.S2-L242H	This paper	N/A
Plasmid:pcDNA3.1.S2-R246I	This paper	N/A
Plasmid:pRP[Exp]-EGFP-CMV.ACE2-human	This paper	N/A
Plasmid:pRP[Exp]-EGFP-CMV.ACE2-mink	This paper	N/A
Plasmid:pRP[Exp]-EGFP-CMV.ACE2-dog	This paper	N/A
Plasmid:pRP[Exp]-EGFP-CMV.ACE2-cat	This paper	N/A
Plasmid:pRP[Exp]-EGFP-CMV.ACE2-pangolin	This paper	N/A
Plasmid:pRP[Exp]-EGFP-CMV.ACE2-pig	This paper	N/A
Plasmid:pRP[Exp]-EGFP-CMV.ACE2-mouse	This paper	N/A
Plasmid:pRP[Exp]-EGFP-CMV.ACE2-bat	This paper	N/A
Plasmid:pRP[Exp]-EGFP-CMV.ACE2-cow	This paper	N/A
Plasmid:pRP[Exp]-EGFP-CMV.ACE2-rabbit	This paper	N/A
Plasmid:pRP[Exp]-EGFP-CMV.ACE2-ferret	This paper	N/A
Plasmid:pRP[Exp]-EGFP-CMV.ACE2-sheep	This paper	N/A
Plasmid:pRP[Exp]-EGFP-CMV.ACE2-civet	This paper	N/A
Plasmid:pRP[Exp]-EGFP-CMV.ACE2-monkey	This paper	N/A

**Software and algorithms**		

GraphPad Prism version 8.0.1(244)	GraphPad Software	https://www.graphpad.com
Microsoft Office Home and Student 2019	Microsoft Corporation	https://www.microsoft.com/microsoft-365
Heatmap Illustrator (HemI) version 1.0.3.7	(Deng et al., 2014)	http://ccd.biocuckoo.org/
BioEdit version 7.2	BioEidt Software	https://bioedit.software.informer.com/
Adobe Illustrator CC 2018	Adobe	https://www.adobe.com
PyMOL	Schrödinger	https://pymol.org/2/

### Resource availability

#### Lead contact

Further information and requests for resources and reagents should be directed to and will be fulfilled by the Lead Contact, Youchun Wang (wangyc@nifdc.org.cn).

#### Materials availability

All unique reagents generated in this study are available from the Lead Contact with a completed Materials Transfer Agreement.

#### Data and code availability

Original data for Figures 2A, 2B, 3, 4A, 4B, 5A-5C, S2, and S3 have been deposited to Mendeley Data: http://dx.doi.org/10.17632/hkg5wjv9ry.2.

### Experimental models and subject details

#### Cell lines

Huh-7 (Japanese Collection of Research Bioresources [JCRB], 0403), Vero (ATCC, CCL-81), LLC-MK2 (ATCC, CCL-7) and HEK293T (American Type Culture Collection [ATCC], CRL-3216) cells were cultured in Dulbecco’s modified Eagle medium (DMEM, high glucose; Hyclone, Logan, UT). All the cells were cultured in media supplemented with 100 U/mL of Penicillin-Streptomycin solution (GIBCO, Germany), 20 mM N-2-hydroxyethyl piperazine-N-2-ethane sulfonic acid (HEPES, GIBCO), and 10% fetal bovine serum (FBS, Pansera ES, PAN-Biotech GmbH, Germany) in a 5% CO_2_ environment at 37°C. Trypsin-EDTA (0.25%, GIBCO) was used to detach cells for subculturing every 2–3 days.

#### Human sera

Sera from 15 convalescent patients were collected from the Chinese CDC of Heilongjiang (CSC1, CSC2, CSC3, CSC4, CSC5, CSC6, CSC7, CSC8, CSC9, CSC10, CSC11, CSC12, CSC13 and CSC14) and Liaoning (CSC15) provinces. A series of 30 convalescence serum samples (NH1.1, NH1.2, NH1.3, NH2.1, NH2.2, NH2.3, NH3.1, NH3.2, NH3.3, NH4.1, NH4.2, NH4.3, NH5.1, NH5.2, NH5.3, NH6.1, NH6.2, NH6.3, NH7.1, NH7.2, NH7.3, NH8.1, NH8.2, NH8.3, NH9.1, NH9.2, NH9.3, NH10.1, NH10.2 and NH10.3) were provided by the University of South China. Written informed consent was obtained from each individual for serum collection.

#### Sera from RBD-immunized mice

The sera were obtained by immunizing nine SPF BALB/c mice with the SARS-CoV-2 RBD protein. RBD protein (20 μg) was mixed with an equal amount of aluminum adjuvant and injected subcutaneously through the head and neck. Immunization was performed once every other week (a total of three times). Blood samples were collected 14 days after the third immunization. Sera of three mice were pooled and labeled as RBD1, RBD2, and RBD3. The protocol of the animal study was approved by the Ethical Review Committee for Animal Welfare of The National Institutes for Food and Drug Control.

### Method details

#### Plasmid construction

The SARS-CoV-2 spike (GenBank: MN908947) expression plasmid was optimized for mammalian codon usage and was inserted into the eukaryotic expression vector pcDNA3.1 using the BamHI and *Xho*I sites to obtain the plasmid pcDNA3.1-SARS-CoV-2-spike (pcDNA3.1.S2).

A total of 14 ACE2 expressing plasmids were constructed, including human (BAB40370.1), mink (QNC68911.1), dog (MT663955), cat (MT663959), pangolin (XP_017505746.1), pig (NP_001116542.1), mouse (ABN80106.1), bat (KC881004.1), cow (NP_001019673.2), rabbit (MT663961), ferret (MT663957), sheep (XP_011961657.1), civet (AY881174.1), and monkey (MT663960). Each gene of the 14 species was mammalian codon-optimized. The codon-optimized ACE2 fused with a FLAG tag (GACTACAAGGACGATGACGATAAG) at the 3′-terminal end was synthesized by General Biol. Inc, (Anhui, China). Each synthesized sequence was inserted into the eukaryotic expression vector pRP[Exp]-EGFP-CMV using the BamHI and *Xho*I sites to get ACE2 expression plasmids from the different species.

#### Site-directed mutagenesis

Based on pcDNA3.1.S2, 18 mutant plasmids were constructed. The point mutation method was conducted as described in our previous study (Nie et al., 2020a, 2020b). Briefly, PCR amplification was performed using the SARS-CoV-2 Spike D614G plasmid as a template. The amplification system and conditions were designed according to the manual of PrimeSTAR (Takara) reagents. The PCR products were digested by *Dpn*I (NEB) overnight and used to transform *E. coli.* DH5α competent cells. The bacteria seeded on the corresponding resistance plates were incubated at 37°C overnight. Single colonies were selected and then sequenced to confirm the integrity of the expected mutation. Specific mutation sites and corresponding primers **(**Sangon Biotech) are shown in the Key Resources Table.

#### Preparation of the ACE2 overexpressing cells

ACE2 expressing cells from different species were prepared as follows: taking the T75 flask as an example, HEK293T cells were transfected with 30 μg of ACE2 plasmid using Lipofectamine 2000 (Invitrogen) transfection reagent to obtain ACE2 overexpressing cells. The culture medium was the same as that used for the HEK293T cells. After 24 h culture in a 5% CO_2_ environment at 37°C, the cell surface expression of the FLAG-tagged ACE2 orthologs was assessed by flow cytometry: 1x10^6^cells/tube were stained with 1 μg/ml PE labeled anti-Flag antibody (Biolegend). The fluorescent signal was examined using a BD FACS CantoTM II Flow Cytometer.

#### Preparation of pseudotyped viruses

The pseudotyped viruses of the SARS-CoV-2 variants and the point mutation pseudotyped viruses were constructed using the methods reported in our previous study (Nie et al., 2020a, 2020b). Briefly, one day prior to transfection for virus production, HEK293T cells were digested and adjusted to a concentration of 5-7 × 10^5^ cells/mL in a 15ml culture medium and incubated overnight in an incubator at 37°C with 5% CO_2_. When cells reached 70%–90% confluence, the culture medium was discarded and 15 mL G^∗^ΔG-VSV virus (VSV G pseudotyped virus, Kerafast) with a concentration of 7.0 × 10^4^ TCID_50_/mL was used for infection. At the same time, 30 μg of the S protein expression plasmid was transfected according to the instructions of Lipofectamine 3000 (Invitrogen), and then the cells were cultured in an incubator at 37°C and 5% CO_2_. After 4-6 hours, the cell medium was discarded, and the cells were gently washed two times with PBS+1% FBS. Next, 15 mL fresh complete DMEM was added to the T75 cell culture flask, which was placed in an incubator at 37°C with 5% CO_2_ for 24 h. After that, the SARS-CoV-2 pseudotyped virus containing the culture supernatant was harvested, filtered, aliquoted, and frozen at −70°C for further use.

#### Quantification of pseudotyped virus particles using RT-PCR

RNA of SARS-CoV-2 pseudotyped virus and point mutation pseudotyped virus was extracted using the QIAamp Viral RNA Mini Kit (QIAGEN, Germany). The virus DNA was obtained by reverse transcription using the SuperScript III First-Strand Synthesis System for RT-PCR kit reagent (Invitrogen). RT-PCR was performed using TB Green Premix Ex TaqII (Takara). The plasmid containing the P protein gene of the VSV virus was used as the standard to calculate the viral copy number. See the primers in the Key Resources Table.

#### Infection assays

After quantification by RT-PCR, the pseudotyped virus was diluted to the same particle number, and 100 μL aliquots were added into 96-well cell culture plates. Cells of the assayed cell lines were then digested with trypsin and added into each well at 2 × 10^4^/100 μl. Chemiluminescence monitoring was carried out after a 24 h incubation with 5% CO_2_ at 37°C. The supernatant was adjusted to 100 μL for each sample. Luciferase substrate was mixed with cell lysis buffer (Perkinelmer, Fremont, CA) and was added to the plate (100 μl/well). Two minutes later, 150 μL of lysate was transferred to opaque 96-well plates. The luminescence signal was detected using a PerkinElmer Ensight luminometer, with data collected in terms of relative luminescence unit (RLU) values. Each group contained two replicates, and these experiments were repeated three times.

#### Neutralization assays

The effects of the monoclonal antibodies and sera on the entry inhibition of the pseudotyped viruses was evaluated by detecting the decrease of luciferase gene expression (Nie et al., 2020b). The samples were serially diluted three times (30 folds as the initial dilution) for a total of seven gradients in the 96 well plates. The virus solution was subsequently added to the wells. Seven virus control wells (without antibody samples) and seven cell control wells (without virus or antibody samples) were included for each 96-well plate. The 96-well plates were incubated at 37°C for 1 h. Huh7 cells were then digested and added to each well (2 × 10^4^/100 μl). After incubation with 5% CO_2_ at 37°C for 24 hours, luminescence was measured as described above. The sample EC_50_ (median effect concentration) was calculated using the Reed-Muench method (Nie et al., 2020b).

#### Structural modeling

We modeled the spike protein based on the Protein Data Bank coordinate set 6VXX, showing the first step of the S protein trimer activation with one RBD domain in the up position, bound to the hACE2 receptor (Walls et al., 2020). We used the Pymol program (The PyMOL Molecular Graphics System, Version 2.2.0, Schrödinger, LLC.) for visualization.

### Quantification and statistical analysis

GraphPad Prism 8 was used for plotting and statistical analysis; the values were expressed as means ± SEM. One-way ANOVA and Holm-Sidak’s multiple comparison tests were used to analyze differences between groups. A p value of less than 0.05 was considered to be significant. ^∗^ p < 0.05, ^∗∗^ p < 0.01, ^∗∗∗^ p < 0.005, ^∗∗∗∗^ p < 0.001.
